# Modulation of MagR magnetic properties via iron–sulfur cluster binding

**DOI:** 10.1038/s41598-021-03344-2

**Published:** 2021-12-14

**Authors:** Zhen Guo, Shuai Xu, Xue Chen, Changhao Wang, Peilin Yang, Siying Qin, Cuiping Zhao, Fan Fei, Xianglong Zhao, Ping-Heng Tan, Junfeng Wang, Can Xie

**Affiliations:** 1 State Key Laboratory of Membrane Biology, Laboratory of Molecular Biophysics, School of Life Sciences, Peking University, Beijing, 100871 China; 2 High Magnetic Field Laboratory, Hefei Institutes of Physical Science, Chinese Academy of Sciences, Science Island, Hefei, 230031 China; 3 State Key Laboratory for Superlattices and Microstructures, Institute of Semiconductors, Chinese Academy of Sciences, Beijing, 100083 China; 4 Department of Microbiology and Biochemistry, Rutgers University, New Brunswick, NJ USA; 5 International Magnetobiology Frontier Research Center, Science Island, Hefei, 230031 China

**Keywords:** Biochemistry, Biophysics, Structural biology, Zoology, Ecology

## Abstract

Iron–sulfur clusters are essential cofactors found in all kingdoms of life and play essential roles in fundamental processes, including but not limited to respiration, photosynthesis, and nitrogen fixation. The chemistry of iron–sulfur clusters makes them ideal for sensing various redox environmental signals, while the physics of iron–sulfur clusters and its host proteins have been long overlooked. One such protein, MagR, has been proposed as a putative animal magnetoreceptor. It forms a rod-like complex with cryptochromes (Cry) and possesses intrinsic magnetic moment. However, the magnetism modulation of MagR remains unknown. Here in this study, iron–sulfur cluster binding in MagR has been characterized. Three conserved cysteines of MagR play different roles in iron–sulfur cluster binding. Two forms of iron–sulfur clusters binding have been identified in pigeon MagR and showed different magnetic properties: [3Fe–4S]-MagR appears to be superparamagnetic and has saturation magnetization at 5 K but [2Fe–2S]-MagR is paramagnetic. While at 300 K, [2Fe–2S]-MagR is diamagnetic but [3Fe–4S]-MagR is paramagnetic. Together, the different types of iron–sulfur cluster binding in MagR attribute distinguished magnetic properties, which may provide a fascinating mechanism for animals to modulate the sensitivity in magnetic sensing.

## Introduction

Iron–sulfur clusters are evolutionarily ancient cofactors participating in many essential biological processes, including but not limited to, respiration, photosynthesis, nitrogen fixation, DNA replication and repair^1–5^. The in vitro chemistry of iron–sulfur clusters has been thoroughly documented through the years. [2Fe–2S], [3Fe–4S], and [4Fe–4S] clusters are the simplest iron–sulfur clusters in nature. Either ferrous (Fe^2+^) or ferric (Fe^3+^) iron from clusters coordinate with proteins through thiolate from cysteine residues in most cases^6–8^. The biogenesis of bacterial iron–sulfur protein could be catalyzed by three different systems^3,6,9^. Iron–sulfur cluster (ISC) and sulfur utilizing factor (SUF) systems are more common in bacteria^6,9^. The nitrogen fixation (NIF) system is dedicated to the maturation of the iron–sulfur proteins related to nitrogen fixation in azototrophic bacteria^3,6,9^. In *E. coli*, the ISC system is utilized under normal growth conditions^10^, while the SUF system is used under iron limitation and oxidative stress conditions^6,11,12^. During the evolutionary process, these two systems were transferred to the mitochondria, cytoplasm, and nucleus of eukaryotes containing iron–sulfur protein through endosymbiosis^4,12–14^. In the eukaryotic cytosol and nuclei, the assembly of iron–sulfur proteins requires the assistance of both the mitochondrial ISC assembly machinery and a mitochondrial ISC export system^15,16^.

The mid-range redox potential of iron–sulfur clusters makes them ideal for sensing various redox environmental signals via electron transport and the mechanism has been extensively studied^1,2,17^, however, essential knowledge regarding the physics of iron–sulfur clusters and their host proteins are lacking. MagR, an A-type iron–sulfur protein originally named IscA1, has been reported as a putative magnetoreceptor^18^. It forms 24 * 15 nm rod-like complex with cryptochrome (Cry) and shows intrinsic magnetic moment of roughly 0.09–0.1 µB/f.u. in vitro^18^. The magnetic property of MagR and MagR/Cry complex have been further confirmed theoretically^19,20^ and experimentally^21–23^. Currently, there are four hypotheses have been proposed to explain the principle of animal magnetoreception: electromagnetic induction model^24–26^, magnetite model^27–32^, radical pair model based on cryptochrome (Cry)^33–40^ and biocompass model based on MagR/Cry complex^18,41^. In biocompass model, the Cry–MagR interaction was nearly abolished by removing the iron–sulfur cluster in MagR^18^, suggesting that the iron–sulfur cluster may be critical for structural stability, or play important roles in the assembly of MagR/Cry complex thus contribute to the ability of magnetosensing. A systematic investigation of the structural and functional features of iron–sulfur cluster binding in MagR may provide insights in the currently unresolved origin of the magnetic moment of MagR/Cry complex, and shed light on our understanding of the mechanism of animal magnetoreception, the least understood sense in biology.

Here, multiple approaches have been applied to characterize the binding property of the iron–sulfur cluster in the pigeon (*Columba livia*) MagR (clMagR). Two different types of the iron–sulfur cluster binding, [2Fe–2S] and [3Fe–4S], in clMagR protein have been identified, and [2Fe–2S]-clMagR severs as an intermediator during [3Fe–4S]-clMagR formation. Mutagenesis studies suggested that three conserved cysteines played different roles in binding different iron–sulfur clusters. Intriguingly, distinguished magnetic features have been observed when clMagR binds different iron–sulfur clusters. [2Fe–2S]-MagR is paramagnetic at 5 K and diamagnetic at 300 K, however, [3Fe–4S]-MagR appears to be superparamagnetic and has saturation magnetization at 2 T about 0.22771 emu/g protein at 5 K, and paramagnetic at 300 K. The data presented in this study extended our understanding of how iron–sulfur binding affects the magnetic feature of MagR and may provide insights to the mechanism of how magnetoreception is regulated in animal navigation.

## Results

### The binding of [2Fe–2S] and [3Fe–4S] in clMagR

**Figure 1:**
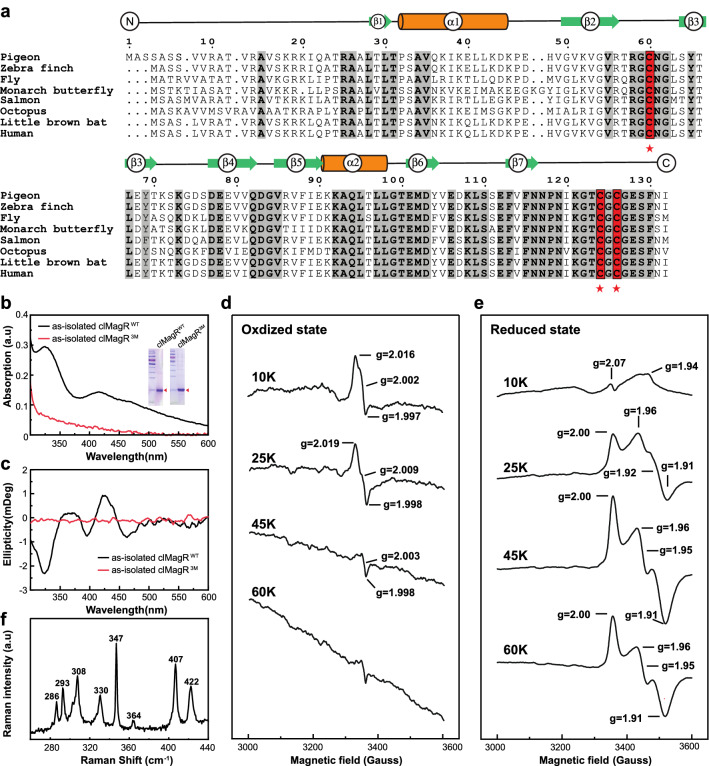
Characterization of iron–sulfur clusters in as-isolated clMagR. (**a**) Sequence alignment of MagR in eight representative species. Predicted secondary structures are shown in the upper lines, with two alpha-helices (orange cylinders) and seven beta-strands (green arrows). Conserved residues with iron–sulfur cluster binding properties are shown in the red background (100% conserved), indicated by stars. Other conserved residues are shown in the gray background and bold fonts. Species’ common name, Latin name and sequence ID in NCBI are listed as follows: Pigeon (*Columba livia*), XP_005508102.1*; Zebra finch(*Taeniopygia guttata*), XP_002194930.1*; Fly(*Drosophila melanogaster*), NP_573062.1*; Monarch butterfly(*Danaus plexippus*), AVZ24723.1*; Salmon(*Salmo salar*), XP_013999046.1*; Octopus(*Octopus bimaculoides*), XP_014786756.1*; Little brown bat(*Myotis lucifugus*), XP_006102189.1*; Human(*Homo sapiens*), NP_112202.2*. (**b**) UV–Vis absorption spectrum of as-isolated pigeon MagR (clMagR^WT^, black) and C60AC124AC126A substitution mutant (clMagR^3M^, red), indicating three cysteines contribute to the iron–sulfur cluster binding. SDS-PAGEs of protein preparation are shown as inserts, theoretical mass of the clMagR monomer and clMagR^3M^ monomer were 16.41 kDa, 16.31 kDa, respectively. (**c**) CD spectrum of as-isolated clMagR^WT^(black) and clMagR^3M^(red). (**d**, **e**) X-band EPR spectrum of as-isolated clMagR^WT^ at oxidized (**d**) and reduced status (**e**). The samples were frozen in TBS buffer and the spectrums were recorded at various temperatures (10 K, 25 K, 45 K, 60 K). (**f**) Low-temperature resonance Raman spectra of as-isolated clMagR^WT^. Spectra were recorded at 17 K using 488 nm laser excitation.

Three conserved cysteines (C60, C124, and C126) of clMagR in a CX_n_CGC sequence motif (n is 63–65 in most cases) play critical roles in iron–sulfur cluster binding^18^ (Fig. 1a), which has been further validated by alanines substitution mutant clMagR^3M^ (C60A, C124A, and C126A mutation of clMagR^WT^). Strep-tagged clMagR^WT^ and clMagR^3M^ were freshly prepared (labeled as “as-isolated”) and purified to homogeneity under aerobic conditions. The clMagR^WT^ protein showed brown color and clMagR^3M^ appeared colorless in the solution, indicating the presence or absence of iron–sulfur cluster, respectively. Consistently, the Ultraviolet–visible (UV–Vis) spectrum of as-isolated clMagR^WT^ showed absorption from 300-to-600-nm region, and with an absorption peak at 325 and 415 nm, and a shoulder at 470 nm, whereas these absorption peaks were abolished in clMagR^3M^ (Fig. 1b). Circular dichroism (CD) spectroscopy was applied to characterize the types of iron–sulfur cluster and their protein environments during cluster maturation^42–44^. As shown in Fig. 1c, clMagR^WT^ shows distinct positive peaks at 371 nm and 426 nm and three negative peaks at 324 nm, 396 nm, and 463 nm, respectively, suggesting the presence of [2Fe–2S] cluster^45^. However, it is worth pointing out that [4Fe–4S] or [3Fe–4S] clusters usually exhibit negligible CD intensity compared to [2Fe–2S] as shown previously in ^Nif^IscA^45,46^, thus CD spectroscopy cannot exclude the existence of [4Fe–4S] or [3Fe–4S]. Electron paramagnetic resonance (EPR) spectroscopy was then used to analyze different states of as-isolated clMagR^WT^. The oxidized clMagR^WT^ was S = 1/2 species, characterized by a rhombic EPR signal with g values at g_1_ = 2.016, g_2_ = 2.002, and g_3_ = 1.997 (Fig. 1d) which disappeared at 45 K, suggesting the presence of [3Fe–4S]^1+^ cluster^47,48^. After reduced with sodium dithionite (Fig. 1e), EPR signal from [2Fe–2S] cluster can be observed until the temperature increased to 60K^49–51^. Thus, two distinct iron–sulfur clusters were assigned by EPR spectroscopy of clMagR^WT^.

Considering some iron–sulfur clusters in proteins are diamagnetic and therefore EPR silent, low-temperature Resonance Raman (RR) spectroscopy was then utilized as a probe to characterize those clusters^52^. With 488 nm excitation, the RR spectra of clMagR^WT^ in the iron–sulfur stretching region (240–450 cm^−1^) show the presence of [3Fe–4S]^1+^ cluster (represented by two bridging modes at 286 and 347 cm^−1^, and one terminal modes at 364 cm^−1^) and [2Fe–2S]^2+^ cluster (represented by three iron–sulfur bridging mode at 293, 308 and 330 cm^−1^ and two terminal modes at 407 and 422 cm^−1^, as shown in Fig. 1f)^52–56^. Taking together, we conclude that as-isolated clMagR^WT^ contains both cystine-ligated [2Fe–2S] cluster and [3Fe–4S] cluster.

### The assembly and conversion of [2Fe–2S] and [3Fe–4S] in clMagR

**Figure 2:**
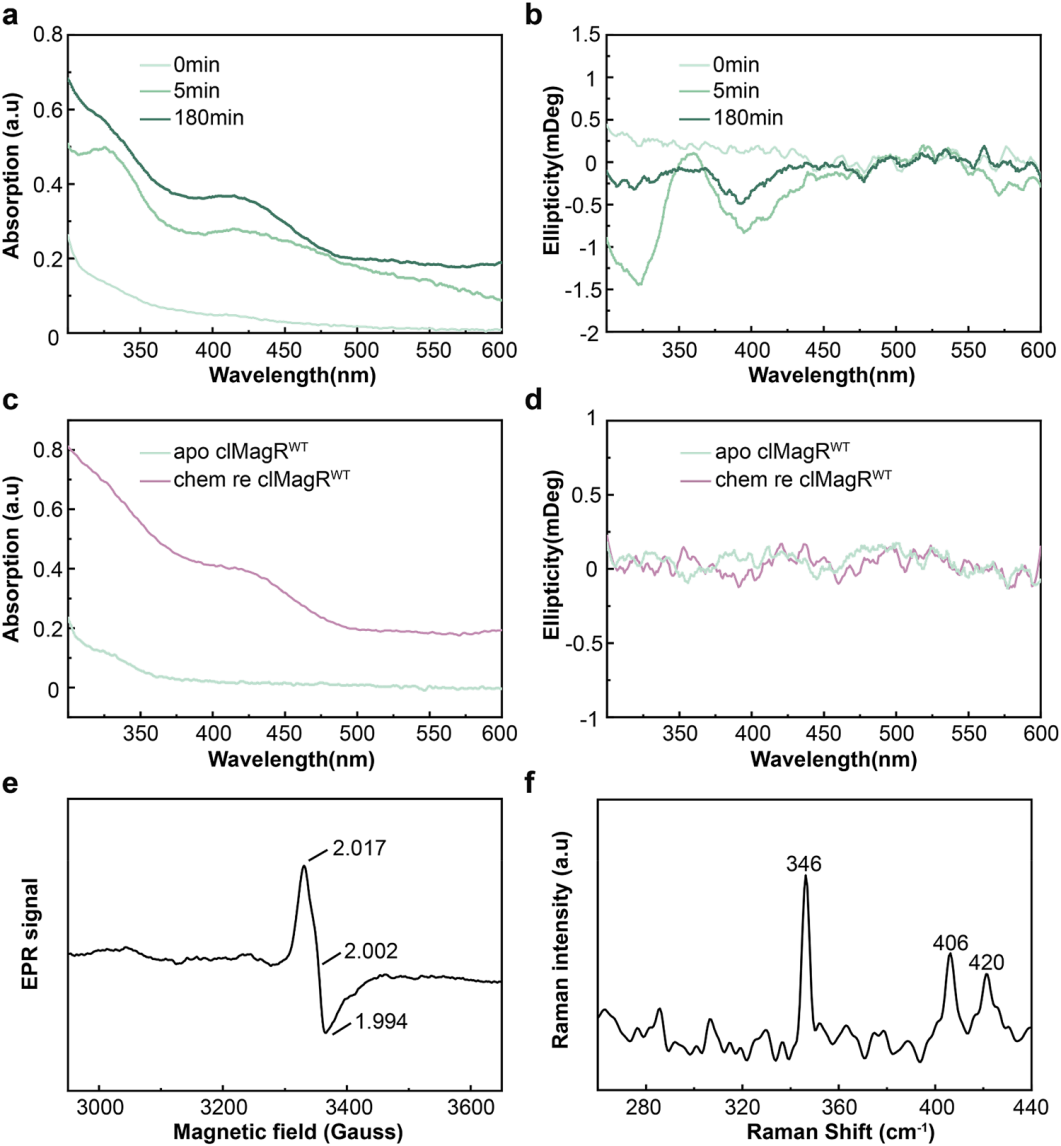
Iron–sulfur cluster assembly on clMagR. (**a**, **b**) IscS-mediated iron–sulfur cluster assembly on clMagR monitored as a function of time by UV–Vis absorption (**a**) and CD spectroscopy (**b**). The spectra shown were taken with samples of pretreated clMagR to remove iron–sulfur clusters before reconstitution (apo-clMagR, 0 min, light green), incubated with IscS after 5 min (green), and after 180 min (dark green). (**c**, **d**) chemical reconstitution-mediated iron–sulfur cluster assembly on clMagR monitored as a function of time by UV–Vis absorption (**c**) and CD spectroscopies (**d**). The spectra shown were taken with samples of pretreated clMagR to remove iron–sulfur clusters before reconstitution (apo-clMagR, light green) and chemically reconstituted clMagR (chem re clMagR, purple). (**e**) X-band EPR spectrum of chemically reconstituted clMagR^WT^. The spectrum was recorded at 10 K. (**f**) Low-temperature resonance Raman spectra of chemically reconstituted clMagR. Protein and reagent concentrations are described in the Methods. Spectra were recorded at 17 K using 488 nm laser excitation.

Iron–sulfur cluster assembly of IscA, an clMagR homology protein in bacteria, is mediated by cysteine desulfurase IscS^2^. To elucidate how iron–sulfur cluster assembles in clMagR, time-course experiment was performed, and UV–Vis absorption and CD spectrum were used to monitor the IscS-catalyzed iron–sulfur cluster assembly in clMagR (Fig. 2). No signal of the iron–sulfur cluster was recorded when the reaction begins (0 min), and then the characteristic visible absorption peak and CD spectrum of clMagR^WT^ appeared after 5 min, indicating [2Fe–2S] cluster assembled. As the reaction proceeds, the UV–Vis absorption intensity increased and after 180 min the signal was dominated by a broad shoulder centered at 415 nm (Fig. 2a). Concomitantly, the CD spectrum of the [2Fe–2S] center decreased and then almost disappeared after 180 min, indicating that [2Fe–2S] had been converted to [3Fe–4S] clusters and the reconstitution finished (Fig. 2b).

Iron–sulfur cluster assembly can be achieved by chemical reconstitution as well, since iron–sulfur apo-proteins are able to spontaneously form iron–sulfur clusters in vitro when supplied with iron and sulfide under reducing conditions^1,43,57^. With this approach, started with apo-clMagR^WT^, we successfully reconstituted [3Fe–4S] cluster in clMagR protein, confirmed by UV–Vis absorption and CD spectrum result (Fig. 2c,d). To further validate if [3Fe–4S] is the sole type of iron–sulfur cluster in clMagR after chemical reconstitution, EPR and low-temperature Resonance Raman spectroscopy were applied (Fig. 2e,f). The chemically reconstituted clMagR^WT^ was S = 1/2 species, characterized by a rhombic EPR signal with g values at g_1_ = 2.017, g_2_ = 2.002, and g_3_ = 1.994 (Fig. 2e). The signal is assigned to a S = 1/2 [3Fe–4S]^1+^ cluster. The Low-temperature Resonance Raman spectrum showed an intense band at 346 cm^−1^ and additional bands at 406 and 420 cm^−1^, which demonstrated that chemically reconstituted clMagR^WT^ only contains [3Fe–4S]^1+^ cluster (Fig. 2f).

**Figure 3:**
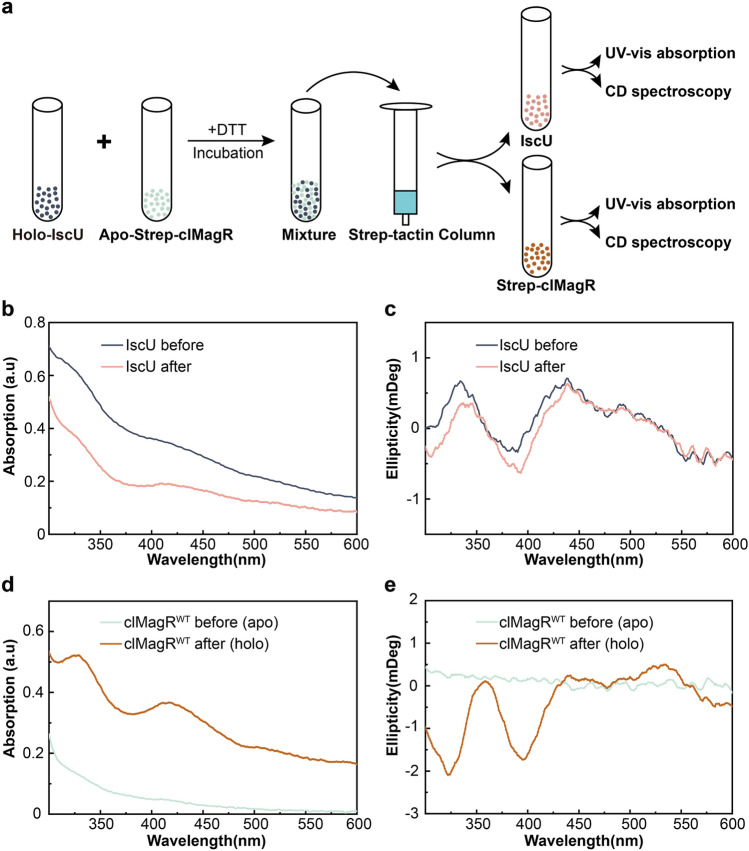
clMagR serve as carrier protein to accept [2Fe–2S] cluster from IscU in vitro. (**a**) A cartoon schematically illustrates the experimental procedures of in vitro iron–sulfur cluster transfer from IscU to clMagR. (**b**, **c**) The UV–Vis absorption (**b**) and CD spectra (**c**) of IscU. IscU protein samples were taken before mixing with apo-clMagR (holo-IscU, black lines) and after incubated with apo-clMagR for 180 min (pink lines). (**d**, **e**) The UV–Vis absorption (**d**) and CD spectra (**e**) of clMagR. clMagR samples were taken before mixing with holo-IscU (apo-clMagR, light green lines) and after incubated with holo-IscU for 180 min (holo-clMagR, brown lines).

We further investigated if clMagR could serve as an iron–sulfur carrier protein to accept [2Fe–2S] cluster from scaffold protein such as IscU^58^. Briefly, 400 µM holo-IscU was mixed with 400 µM strep-tagged apo-clMagR^WT^ and incubated for 180 min under reduced condition, then, after desalting and strep-tactin affinity column separation, UV–Vis absorption and CD spectroscopy were applied the iron–sulfur cluster transfer process (Fig. 3a). The intensity of UV–Vis spectrum decreased in IscU (Fig. 3b) but significantly increased in clMagR after reaction (Fig. 3d), indicating [2Fe–2S] cluster was transferred from IscU to clMagR^59^. Consistently, CD spectrum of IscU and clMagR also confirmed that [2Fe–2S] transfer occurred between IscU and clMagR (Fig. 3c,e). The resulting spectrum is very similar to that of the [2Fe–2S] intermediate assembled on IscS mediated reconstituted apo-clMagR (Fig. 2b).

### Cys-60 is essential for clMagR to bind [3Fe–4S] cluster, not [2Fe–2S] cluster

Three conserved cysteines (C60, C124, and C126) of clMagR play critical roles in iron–sulfur cluster binding, and the substitute mutation of these three residues abolished iron–sulfur binding (Fig. 1b,c)^18^. To elucidate if three cysteines bind [2Fe–2S] and [3Fe–4S] differently, single Cys-to-Ala substitutions (C60A, C124A, and C126A) were made and their iron–sulfur binding properties were characterized.

**Figure 4:**
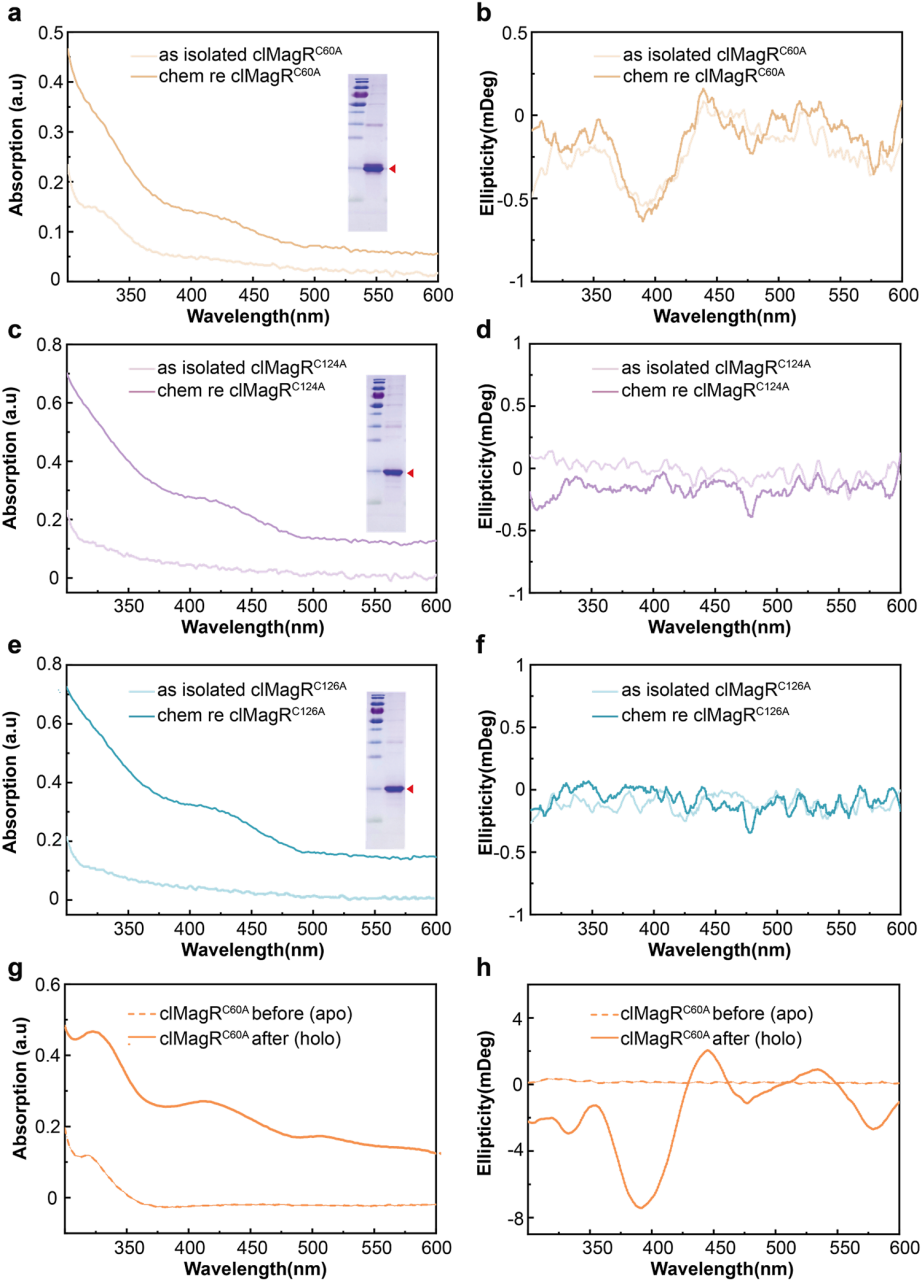
Three conserved cysteines play different roles in iron–sulfur binding in clMagR. (**a**, **b**) Chemical reconstitution-mediated iron–sulfur cluster assembly on apo-clMagR^C60A^ monitored by UV–Vis absorption (**a**) and CD spectroscopies (**b**). The samples of spectra shown are as-isolated clMagR^C60A^ (light orange) and chemically reconstituted clMagR^C60A^ (chem re clMagR^C60A^, orange). (**c**, **d**) chemical reconstitution-mediated iron–sulfur cluster assembly on clMagR^C124A^ monitored by UV–Vis absorption (**c**) and CD spectroscopies (**d**). The samples of spectra shown are as-isolated clMagR^C124A^ (light purple) and chemically reconstituted clMagR^C124A^ (chem re clMagR^C124A^, purple). (**e**, **f**) chemical reconstitution-mediated iron–sulfur cluster assembly on pigeon clMagR^C126A^ monitored by UV–Vis absorption (**e**) and CD spectroscopies (**f**). The samples of spectra shown are as-isolated clMagR^C126A^ (light blue) and chemically reconstituted clMagR^C126A^ (chem re clMagR^C126A^, blue). SDS-PAGE results were shown in the right of corresponding UV–Vis spectra as inserts (**a**, **c**, **e**). The theoretical mass of the clMagR^C60A^ monomer, clMagR^C124A^ monomer and clMagR^C126A^ monomer were 16.38 kDa. (**g**, **h**) The UV–Vis absorption (**c**) and CD spectra (**d**) of clMagR^C60A^ obtained by mixing apo-clMagR^C60A^ and holo-IscU which was recorded before the addition of apo-clMagR^C60A^ (dotted orange lines) and after incubation with apo-clMagR^C60A^ for 180 min (orange lines). Protein and reagent concentrations are described in the Experimental procedures.

Freshly purified as-isolated clMagR^C60A^ showed light brown color, and [2Fe–2S] cluster binding was verified by UV–Vis absorption and CD spectrum (Fig. 4a,b). A typical protein-bound [2Fe–2S] cluster absorption peak at 325 nm and a shoulder at 415 nm are visible in UV–Vis absorption (Fig. 4a, light orange line). Consistently, the CD spectrum of as-isolated clMagR^C60A^ mutant had a negative peak at 397 nm and a positive peak at 451 nm (Fig. 4b, light orange line), confirmed the [2Fe–2S] cluster binding, similar to clMagR^WT^. However, in contrast to clMagR^WT^, chemical reconstitution failed to convert [2Fe–2S] cluster to [3Fe–4S] cluster in clMagR^C60A^. As shown in Fig. 4a,b (orange line), chemically reconstituted clMagR^C60A^ showed similar and characteristic [2Fe–2S] UV–Vis absorption peaks and CD spectrum, but not [3Fe–4S] (Fig. 4a,b, orange lines), suggesting that C60A mutation abolished [3Fe–4S] cluster binding ability in clMagR.

In contrast, purified as-isolated clMagR^C124A^ and clMagR^C126A^ were colorless, and the binding of iron–sulfur clusters was barely detectable by UV–Vis and CD spectrum (Fig. 4c–f, light purple, and light blue lines, respectively). However, chemical reconstitution successfully reconstituted [3Fe–4S] cluster binding in both clMagR^C124A^ and clMagR^C126A^ (Fig. 4c–f, purple and blue lines, respectively). After chemical reconstitution, the UV–Vis absorption of both clMagR^C124A^ and clMagR^C126A^ mutants showed the signal of iron–sulfur cluster binding (Fig. 4c,e). Parallel CD spectrum studies confirmed both chemically reconstituted clMagR^C124A^ and clMagR^C126A^ have [3Fe–4S] cluster binding (Fig. 4d,f), similar to chemically reconstituted clMagR^WT^. The results demonstrated that Cys-124 and Cys-126 in clMagR play important roles in [2Fe–2S] cluster binding, thus, mutating these two residues lead to clMagR favors [3Fe–4S] binding.

Considering clMagR can act as a carrier protein to accept iron–sulfur cluster from IscU (Fig. 3), it is worth testing if three cysteines play a different role in this process as well. Holo-IscU was mixed with apo-clMagR single cysteine mutants in a reduced state for 180 min. The apo status of all three mutants (labeled as apo-clMagR^C60A^, apo-clMagR^C124A^, and apo-clMagR^C126A^) had no iron–sulfur cluster binding before mixing with holo-IscU, as shown by negligible UV absorption and CD intensities (Fig. 4g,h and Supplementary Fig. 1a–d, dotted lines). After incubation with holo-IscU and separation of IscU and clMagR mutants, clMagR^C60A^ showed distinct changes in UV–Vis absorption and CD spectrum (Fig. 4g,h). The UV–Vis absorption increased and showed better-resolved peaks at 322 nm, 410 nm, 504 nm (Fig. 4g, orange line), and parallel CD spectra had distinct positive peaks (319 nm, 355 nm, 445 nm, and 534 nm) and four negative peaks (333 nm, 392 nm, 477 nm, and 579 nm, Fig. 4h), indicating [2Fe–2S] cluster was transferred from IscU to clMagR^C60A^. Interestingly, clMagR^C124A^ and clMagR^C126A^ could also accept [2Fe–2S] cluster transferred from holo-IscU, though the binding efficiency is much lower than clMagR^WT^ and clMagR^C60A^, as verified by UV–Vis and CD spectrum (Supplementary Fig. 1a–d). It seems that clMagR^C60A^ accept [2Fe–2S] cluster from scaffold protein IscU more effectively compared with clMagR^C124A^ and clMagR^C126A^. And after incubation with clMagR mutants, UV–Vis absorption of IscU significantly decreased, confirmed that iron–sulfur cluster transfer occurred in between holo-IscU and three clMagR mutants (Supplementary Fig. 1e).

Again, our data demonstrated that three conserved cystines of clMagR played different roles on the iron–sulfur cluster binding, and especially Cys-60 is essential for clMagR to bind [3Fe–4S] cluster, not [2Fe–2S] cluster. Therefore, it is possible to obtain a [2Fe–2S] cluster binding only clMagR by mutating Cys-60. Thus, we labeled clMagR protein samples based on their iron–sulfur cluster in later experiments. For example, we labeled the chemically reconstituted clMagR^WT^ as [3Fe–4S]-clMagR^WT^, and clMagR^C60A^ that accepted [2Fe–2S] cluster from holo-IscU as [2Fe–2S]-clMagR^C60A^, to investigate the magnetic property of clMagR when it binds different iron–sulfur clusters.

### [3Fe–4S]-clMagR shows different magnetic properties from [2Fe–2S]-clMagR

MagR has been reported as a putative magnetoreceptor and exhibits intrinsic magnetic moment experimentally and theoretically when forms complex with cryptochrome (Cry)^18,20,21^. To elucidate if different iron–sulfur clusters binding in clMagR have different magnetic features and respond to external magnetic fields differently, we obtained [3Fe–4S] and [2Fe–2S] bound only clMagR protein by chemical reconstitution of clMagR^WT^ (as [3Fe–4S]-clMagR^WT^) and holo-IscU incubated and re-purified clMagR^C60A^ (as [2Fe–2S]-clMagR^C60A^), respectively, and measured the magnetic moment of these proteins with Superconducting Quantum Interference Device (SQUID) magnetometry. SQUID is a highly sensitive magnetometry to measure extremely subtle magnetic fields and to study the magnetic properties of a range of samples, including extremely low magnetic moment biological samples. Therefore, it has been regularly used as a first test to identify the specific kind of magnetism of a given specimen, such as ferromagnetic, antiferromagnetic, paramagnetic or diamagnetic, by measuring at different temperatures and external magnetic field strength. For example, B-DNA was identified as paramagnetic under low temperature by SQUID^60^.

**Figure 5:**
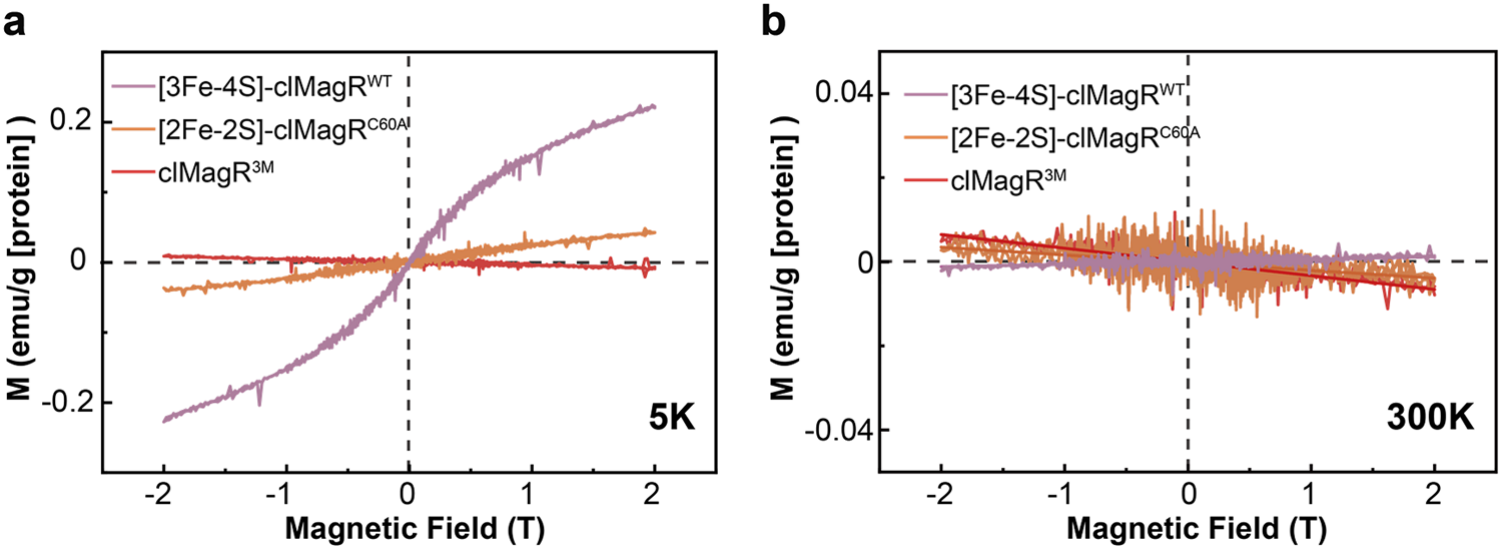
[3Fe–4S]-clMagR^WT^ shows different magnetic properties from [2Fe–2S]-clMagR^C60A^. (**a**) Field-dependent magnetization curves (MH) at 5 K for [2Fe–2S]-clMagR^C60A^ (orange), [3Fe–4S]-clMagR^WT^ (chem re clMagR^WT^, purple), and clMagR^3M^ (red). The magnetic susceptibility of [2Fe–2S]-clMagR^C60A^ is 2.27749E−6 and the magnetic susceptibility of clMagR^3M^ is − 4.0438E−7. (**b**) Field-dependent magnetization curves (MH) at 300 K for [2Fe–2S]-clMagR^C60A^ (orange), [3Fe–4S]-clMagR^WT^ (chem re clMagR^WT^, purple), and clMagR^3M^ (red). And the magnetic susceptibility is − 1.83638E−7, 5.93483E−8, − 3.26432E−7, respectively.

Purified clMagR^3M^ was utilized as a control since it had no iron–sulfur cluster binding due to lack of cysteine residues (Fig. 1b,c). The magnetic measurement was done at different temperatures (5 K and 300 K) and MH curves (magnetization (M) curves measured versus applied fields (H)) were generated for three proteins to reflect the protein magnetic anisotropy. The MH curves of clMagR^3M^ clearly exhibited diamagnetic property at both 5 K and 300 K, suggesting that magnetism of clMagR is dependent on the iron–sulfur cluster (Fig. 5a,b, red lines). In contrast, [3Fe–4S]-clMagR^WT^ showed superparamagnetic behavior at 5 K which has saturation magnetization (M_S_) at 2 T about 0.22771 emu/g protein (Fig. 5a, purple line), [2Fe–2S]-clMagR^C60A^ is paramagnetic at 5 K (Fig. 5a, orange line). Interestingly, at higher temperature such as 300 K, [2Fe–2S]-clMagR^C60A^ is diamagnetic while [3Fe–4S]-clMagR^WT^ is paramagnetic (Fig. 5b, orange line and purple line). The different magnetism, as well as the different saturation magnetization of clMagR with different iron–sulfur binding, are clearly important features of this putative magnetoreceptor, and worth further investigation and validation in vivo in the future.

## Discussion

MagR (IscA), an A-type iron–sulfur protein MagR (IscA1), has been proposed as a candidate magnetoreceptor in the biocompass model^18^. Twenty MagR helically assembles as a rod-like polymer, surrounded by photo-sensitive cryptochrome (Cry), shows magnetic moment of roughly 0.09–0.1 µB/f.u in vitro. However, the mechanism of MagR/Cry complex to respond to external magnetic fields remain largely unknown. The characterization of iron-cluster binding of MagR would provide us clues to understand the currently unresolved mechanism.

MagR (IscA) is a highly conserved iron–sulfur protein widely distributed across all major phyla. The characterization of iron–sulfur cluster binding in MagR in homing pigeon has not been fully investigated yet. Anaerobically purified of *Azotobacter vinelandii* ^Nif^IscA was containing one [2Fe–2S] cluster per homodimer, while NifS-mediated reconstituted ^Nif^IscA contains [4Fe–4S] cluster^46^. As-isolated yeast IscA1 was an apo-protein that could bind an [2Fe–2S] only after chemical reconstitution^59^. More recently, both mouse IscA1 and IscA2, as-isolated (without chemical reconstitution), are [2Fe–2S] proteins^58^. Bianci et al. isolated anaerobically human IscA2 in a [2Fe–2S] form, however, IscA1 was shown to bind [2Fe–2S] cluster only after chemical reconstitution^61^. Here in this study, we present data for the first time that under aerobic conditions, as-isolated pigeon clMagR contains both [2Fe–2S] cluster and [3Fe–4S] cluster without chemical reconstitution, but it only binds [3Fe–4S] cluster after reconstitution. The time-course experiment of IscS-catalyzed iron–sulfur cluster assembly in clMagR demonstrated that [2Fe–2S]-clMagR appears to be an intermediator of [3Fe–4S]-clMagR. The co-existence of different clusters in iron–sulfur proteins has been revealed previously, such as Dre2 from yeast and AaFd^49,55^, but not in A-type iron–sulfur protein such as MagR.

Three fully conserved cysteines (Cys-60, Cys-124, and Cys-126) form the iron–sulfur cluster binding site in MagR. Mutating these three cysteines, as shown in clMagR^3M^, totally abolish iron–sulfur cluster binding. Spectroscopic studies on clMagR mutants demonstrated that three cysteines play different roles. Cys-124 and Cys-126 are ligands for the [2Fe–2S], and Cys-60 is essentially required for [3Fe–4S] binding. Mutating Cys-60 leads to clMagR can only bind [2Fe–2S], and Cys-60 together with Cys-124 or Cys-126 forms a complete [3Fe–4S] cluster binding site. Apparently, the cystine coordination of pigeon clMagR is different from that of its human homologous protein, IscA2^62^. Among three conserved Cysteines (Cys-79, Cys-144, Cys-146) of human IscA2, Cys-79 was essential for the binding of any type of iron–sulfur cluster, while Cys-144 and Cys-146 are both required to bind [4Fe–4S] cluster^62^.

Both [2Fe–2S]-clMagR and [3Fe–4S]-clMagR are paramagnetic at 5 K but [3Fe–4S]-MagR has saturation magnetization which is more like a superparamagnetic protein. While at 300 K, MagR with different iron–sulfur bound forms show distinguished magnetic features. [2Fe–2S]-MagR are diamagnetic but [3Fe–4S]-MagR is paramagnetic. In contrast to the well-documented redox state of iron–sulfur clusters and their host proteins^8^, how such clusters modulate the host protein’s magnetic property remains unknown. Our in vitro study may provide insights to the possibility of magnetic sensing mediated by iron–sulfur protein MagR in pigeon. Considering the possible iron-binding capacity of MagR, as demonstrated by studies on IscA^63,64^, the paramagnetic or superparamagnetic property of MagR could be further boosted under certain circumstances. However, further investigation including magnetic measurement of MagR in vivo certainly would be required in the future.

Taking together, the characterization and identification of two forms of iron–sulfur cluster binding in pigeon MagR, and the observed distinguished magnetic features when MagR hosts different iron–sulfur clusters, suggested a possible dedicated regulatory mechanism of animal magnetoreception. Animals may utilize different iron–sulfur clusters as a magnetic switch to modulate the magnetic property and sensitivity of its magnetoreceptor during navigation. The study presented here extended our understanding of MagR’s functional roles not only as an iron–sulfur protein but also as a candidate magnetoreceptor.

## Method

### Protein expression and purification

The expression vector containging MagR gene of the homing pigeon was constructed as described previously (Qin, *Nature Materials*, 2016), and the genes of clMagR^C60A^, clMagR^C124A^, clMagR^C126A^, and clMagR^3M^ were synthesized and cloned into the expression vector and expressed in *E. coli* strain BL21 (DE3), respectively. Bacteria cells were harvested after induction with 20 µM isopropyl -D-1-thiogalactopyranoside (IPTG) overnight at 288 K. And then lysed by sonication on ice and resuspended in lysis buffer (20 mM Tris, 500 mM NaCl, pH 8.0) with complete protease inhibitor cocktail. After centrifugation, the supernatant was collected and loaded onto the Strep-Tactin affinity column (IBA). The column was washed about 20 column volumes (CV) with buffer W (20 mM Tris, 500 mM NaCl, pH 8.0) to remove unbound proteins. After washing, clMagR protein or its mutants were eluted from the Strep-Tactin affinity columns using buffer E (20 mM Tris, 500 mM NaCl, 5 mM desthiobiotin, pH 8.0). For all SDS-PAGEs, PageRuler Prestained Protein Ladder (Thermo Scientific, Product# 26616) was used as the molecular weight standards.

IscU sequence was obtained from NCBI (https://www.ncbi.nlm.nih.gov/gene/947002) and synthesized, then cloned into expression vector as mentioned previously with a His-tag fused on the N-terminal, and expressed in *E. coli* strain BL21 (DE3). Bacteria cells were harvested after induction with 20 µM IPTG overnight at 288 K, and then lysed by sonication on ice and resuspended in lysis buffer (20 mM Tris, 150 mM NaCl, 10 mM Imidazole, pH 8.0) with complete protease inhibitor cocktail. After centrifugation, the supernatant was collected and loaded onto the Ni–NTA affinity column (QIAGEN). The column was washed about 20 column volumes (CV) with buffer W (20 mM Tris, 150 mM NaCl, pH 8.0) to remove unbound proteins. After washing the matrix, proteins were eluted from the Ni–NTA matrix using elution buffer (20 mM Tris, 150 mM NaCl, 300 mM Imidazole, pH 8.0).

The expression vector of IscS (pET 28-IscS) was a gift from Dr. Huangeng Ding’s Lab. Bacteria cells were harvested after induction with 400 µM IPTG overnight at 288 K, and then lysed by sonication on ice and resuspended in lysis buffer (20 mM Tris, 500 mM NaCl, 10 mM Imidazole, pH 8.0) with complete protease inhibitor cocktail. After centrifugation, the supernatant was collected and loaded onto the Ni–NTA affinity column (QIAGEN). The column was washed about 20 column volumes (CV) with buffer W (20 mM Tris, 500 mM NaCl, pH 8.0) to remove unbound proteins. After washing, IscS protein was eluted from the Ni–NTA matrix using elution buffer (20 mM Tris, 500 mM NaCl, 300 mM Imidazole, pH 8.0). Protein concentration was estimated using a nanodrop spectrophotometer (Thermo Fisher Scientific) with the target protein MW and coefficient of molar extinction ε retrieved from protparam tool website (https://web.expasy.org/protparam/).

### Spectroscopic studies on iron–sulfur cluster binding in MagR

Different spectroscopic approaches were applied to study the iron–sulfur cluster binding in MagR: UV–visible (UV–Vis) absorption, Circular Dichroism (CD), Electron Paramagnetic Resonance (EPR), and low-temperature Resonance Raman (RR) spectroscopy.

UV–Vis absorption measurements in the near UV visible wavelength (300–600 nm) were routinely performed using a nanodrop spectrophotometer (Thermo Fisher Scientific, NanoDrop 2000).

Circular dichroism (CD) is a classic and robust method to either evaluate secondary structures in protein in the far UV range (190 to 260 nm) or to monitor protein-bound co-factors such as metals or iron–sulfur clusters in the near UV–visible range (300 to 600 nm) (Kelly, *Current Protein and Peptide Science*, 2000). Purified wild-type MagR protein and mutants were prepared at 4 mg/mL in TBS buffer (20 mM Tris, 150 mM NaCl, pH 8.0) and were measured in Circular Dichroism Spectrometer MOS-500 (Biologic) at room temperature in 1 cm path quartz cells. Buffer was used as blank control. Data were shown using the ellipticity value in mDeg as measure by the spectrometer with blank subtracted.

X-band (∼ 9.6 GHz) EPR spectra were recorded using EMX plus 10/12 spectrometer (Bruker, Billerica, MA), equipped with Oxford ESR-910 Liquid helium cryostat. Briefly, 1 mM oxidized clMagR (as-isolated clMagR) and 1 mM chemically reconstituted clMagR (chem re clMagR) in TBS buffer (20 mM Tris, 150 mM NaCl, pH 8.0) in a total volume of 0.2 mL mixed with 0.05 mL Glycerol were used, respectively. Reduced clMagR was obtained by adding 10 mM Na_2_S_2_O_4_ into clMagR protein samples. Then, the protein samples were transferred into a 4 mm diameter quartz EPR tube (Wilmad 707-SQ-250 M) and frozen in liquid nitrogen. EPR signals of oxidized clMagR and reduced clMagR were recorded at various temperatures (10 K, 25 K, 45 K, and 60 K). Parameters for recording the EPR spectra were typically 2 G modulation amplitude, 9.40 GHz microwave frequency, and 2 mW incident microwave power, sweep time was 19.2 s.

For low-temperature resonance Raman (RR) spectra, purified proteins were concentrated to ∼ 1 mM and frozen by lyophilization and placed on the surface of a Si wafer chip with a 90-nm-thick SiO2 on the top sealed in a helium-cooled cryogenic station (Montana Instruments) at 17 K. The RR spectra were collected in back-scattering geometry using a Jobin–Yvon HR800 system equipped with a liquid nitrogen cooled charge-coupled detector. The excitation wavelength is 488 nm from an Ar+ laser and a grating with groove density of 1800/mm was used to achieve a spectral resolution of 0.53 cm^−1^. A long working distance 50× objective was used to ensure a high signal-to-noise ratio of the measured Raman spectra.

### IscS-mediated iron–sulfur cluster assembly on clMagR

Apo-clMagR was prepared by pretreating as-isolated clMagR in TBS buffer (20 mM Tris, 500 mM NaCl, pH 8.0) with 10 mM EDTA and 10 mM sodium hydrosulfite overnight. Then the mixture was desalted by desalting column PD-10 (GE Healthcare, 17085101), and the protein obtained was labeled as “Apo-clMagR”.

For IscS-mediated iron–sulfur cluster assembly experiment, Apo-clMagR (as mentioned above) was incubated with IscS (1 µM), ferrous ammonium sulfate (1.6 mM), l-cysteine (20 mM), and DTT (5 mM) in the same buffer for 180 min at room temperature. UV–Vis absorption and CD studies of the time course of iron–sulfur cluster assembly were carried out by measuring the spectra at different time intervals on one sample at room temperature. Briefly, before the reaction begins, the 20% of the reaction mixture was taken out and desalted, labeled as sample after “0 min”. Another 20% of the reaction mixture was taken out after 5 min, desalted, and labeled as sample after “5 min”. Then, the remaining reaction mixture was desalted after 180 min, and labeled as sample after “180 min”. Purification of cluster-bound clMagR to remove excess reagents was achieved by loading the reconstitution mixture onto the desalting column (PD 10, GE Healthcare, 17085101) pre-equilibrated with buffer (20 mM Tris, 150 mM NaCl, pH 8.0) and eluted with buffer (20 mM Tris, 150 mM NaCl, pH 8.0).

For chemical reconstitution experiment, apo-clMagR was incubated with chemical reconstitution mixture (1 mM ferrous ammonium sulfate, 1 mM sodium sulfide, and 5 mM DTT) for 180 min at room temperature. Then, desalted to isolate the clMagR protein from reaction mixture using desalting column PD-10 (GE Healthcare, 17085101), and labeled the protein sample as “chem re clMagR”.

### In vitro iron–sulfur cluster transfer

Holo-IscU was obtained by chemically reconstituted as-isolated IscU. Briefly, as-isolated IscU was incubated with reconstitution mixture (1 mM ferrous ammonium sulfate, 1 mM sodium sulfide, and 5 mM DTT) for 180 min at room temperature. Then desalted to isolate the protein from reaction mixture using desalting column PD-10 (GE Healthcare, 17085101), and and labeled as “Holo-IscU” in Fig. 3a.

The experimental procedure of iron–sulfur transfer between clMagR and holo-IscU was illustrated in Fig. 3a. Briefly, strep-tagged apo-clMagR protein (400 µM) was incubated with holo-IscU (400 µM) in TBS buffer (20 mM Tris, 150 mM NaCl, pH 8.0) after reduced with 5 mM DTT for 2 h. After reaction, two proteins were then separated by loading the reaction mixture contains strep-tagged clMagR and non-tagged IscU to a strep-tactin affinity column pre-equilibrated in buffer (20 mM Tris, 150 mM NaCl, pH 8.0). IscU protein was collected from flow-through, and clMagR protein was then eluted from strep-tactin column with buffer E (20 mM Tris, 150 mM NaCl, 5 mM desthiobiotin, pH 8.0). After reaction and separation, both clMagR and IscU proteins were analyzed for their iron–sulfur-cluster properties by UV–Vis spectrum and CD spectrum as described above to validate the iron–sulfur cluster transfer.

### Magnetic measurements

Protein samples (in TBS buffer contains 20 mM Tris, 150 mM NaCl, pH 8.0) and buffer control (20 mM Tris, 150 mM NaCl, pH 8.0) were lyophilized by freezer dryer (Heto PowerDry LL3000, ThermoFisher) respectively. Magnetic measurements were performed on lyophilized samples (of mass ≈ 4 mg) using a magnetometer (Quantum Design MPMS-3) equipped with a SQUID sensor at different temperatures (5 K and 300 K). The fields applied in the present study were between − 2 and 2 T for samples. MH curves (magnetization (M) curves measured versus applied fields (H)) of proteins were obtained after subtracted the background from buffer control.

## Supplementary Information

Supplementary Information.

## Data Availability

The datasets generated during and/or analyzed during the current study are available from the corresponding author on reasonable request.
