# Supplementary material for: Modulation of MagR magnetic properties via iron–sulfur cluster binding
Source: Sci Rep. 2021 Dec 14;11:23941. doi: 10.1038/s41598-021-03344-2 (PMC8671422; doi:10.1038/s41598-021-03344-2)
Supplement: Supplementary file 1 — Supplementary Information. [file 41598_2021_3344_MOESM1_ESM.docx]

Supplementary data for

**Modulation of MagR Magnetic Properties via Iron-Sulfur Cluster Binding**

Zhen Guo^1^, Shuai Xu^2^, Xue Chen^3^, Changhao Wang^2^, Peilin Yang^1^, Siying Qin^1^, Cuiping Zhao^4^, Fan Fei^2^, Xianglong Zhao^2^, Ping-Heng Tan^3^, Junfeng Wang^2,5^ & Can Xie^1,2,5,*^

*^1^State Key Laboratory of Membrane Biology, Laboratory of Molecular Biophysics, School of Life Sciences, Peking University, Beijing 100871, China*

*^2^High Magnetic Field Laboratory, Hefei Institutes of Physical Science, Chinese Academy of Sciences, Science Island, Hefei, 230031, China*

*^3^State Key Laboratory for Superlattices and Microstructures, Institute of Semiconductors, Chinese Academy of Sciences, Beijing 100083, China*

*^4^Department of Microbiology and Biochemistry. Rutgers University, New Brunswick, NJ, USA*

*^5^International Magnetobiology Frontier Research Center, Science Island, Hefei, 230031, China*

*^*^To whom correspondence and requests for materials should be addressed. E-mail: C.X. (*[*canxie@hmfl.ac.cn*](mailto:canxie@hmfl.ac.cn)*)*


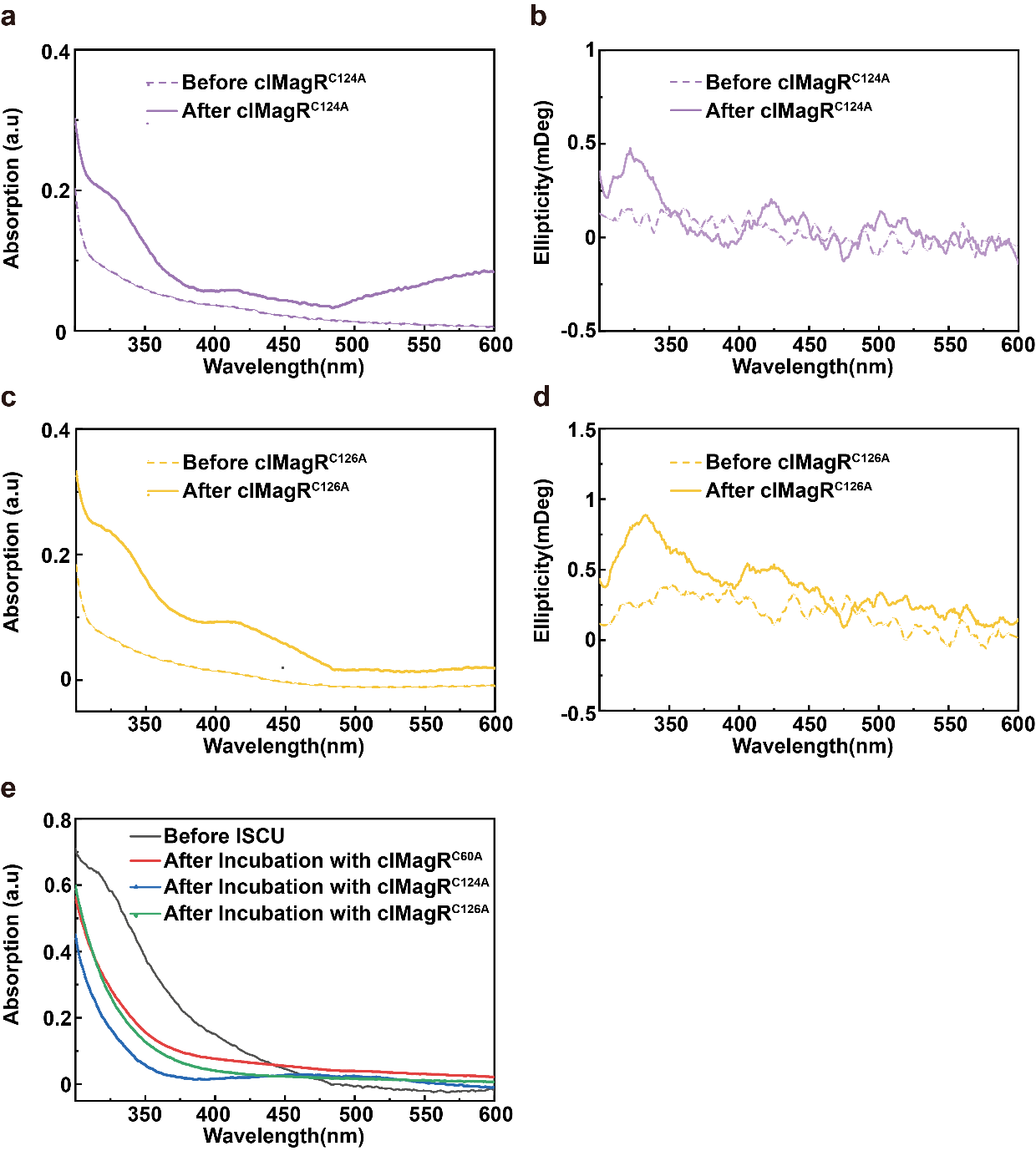


**Supplementary Figure 1. clMagR^C124A^ and clMagR^C126A^ can accept [2Fe-2S] cluster from IscU *in vitro.***

**a, b,** The UV-vis absorption (**a**) and CD spectra (**b**) of clMagR^C124A^ obtained before mixing with holo-IscU (dotted purple lines) and after incubation with IscU for 180 min (purple lines). **c, d,** The UV-vis absorption (**c**) and CD spectra (**d**) of clMagR^C126A^ obtained before mixing with holo-IscU (dotted yellow lines) and after incubation with IscU for 180 min (yellow lines). **e,** The UV-vis absorption of IscU obtained before mixing with apo-clMagR single cysteine mutants (black lines) and after incubation with apo-clMagR single cysteine mutants for 180 min. Protein and reagent concentrations are described in the Experimental procedures.
